# Why Do Individuals Engage in In-Play Sports Betting? A Qualitative Interview Study

**DOI:** 10.1007/s10899-020-09968-9

**Published:** 2020-08-13

**Authors:** Elizabeth A. Killick, Mark D. Griffiths

**Affiliations:** International Gaming Research Unit, Psychology Department, Nottingham Trent University, Nottingham, UK

**Keywords:** Sports betting, In-play betting, Thematic analysis, Live action betting

## Abstract

Increasing technological advancements and changing consumer behavior has resulted in individuals having access to a wider range of online gambling markets and sporting events than ever before. Sports betting in real time has been aided by the accessibility of smartphone devices. Consequently, the popularity of live sports betting (i.e., ‘in-play’ betting) has spread across Europe and around the rest of world. The aim of the present exploratory study was to examine attitudes and opinions towards online sports betting. Qualitative interviews were conducted with 17 males and 2 females aged between 21 and 32 years. Participants were asked a range of semi-structured interview questions based on pre-determined topic areas. Socio-demographic data were collected and the Problem Gambling Severity Index (PGSI) was used to assess problem gambling. The data were analyzed using thematic analysis in order to identify themes. Analysis of the transcripts identified several notable areas including the ease of engaging in in-play sports betting, motivations for engaging in in-play sports betting (including increased excitement, demonstrating knowledge/skill and response to live odds), and different reasons for using the ‘cash-out’ feature. The findings will contribute to the design of future research investigating in-play sports betting behaviours.

## Introduction

In recent years there have been many changes in the way that consumers behave and interact with gambling products. There is a continuous stream of technological development and new features being introduced to the gambling market, especially in the UK sports betting market. Mobile technology has been paramount in contributing to the rise in popularity of online sports betting due to that fact that it provides an easy and accessible method of placing sports bets. Traditionally, sports betting took place inside of bookmakers. Now, due to technological change, sports betting can take place online via smartphones, laptops, and tablets in real time and has altered how individuals can place their bets.

In addition, the number of smartphone users has been increasing over the last few years in the UK and over 85% of adults now own a smartphone (Lee & Paul, 2018). According to the UK Gambling Commission, almost 30% of online gamblers are using a mobile device to place their bets, and there has been a 10% increase in mobile usage between 2016 and 2017 (Gambling Commission, 2018a, b, c). Mobile betting allows individuals the capability to bet from almost any location and can also enable individuals to place a range of live bets on different sporting markets. These bets can be made from numerous locations (e.g., work, home, bars, restaurants) with friends or alone.

There has been an increasing conversion of sports betting into an online activity and this increase has been mirrored by a rise in in-play betting. In-play sports betting comprises the wagering of money on something within a sporting event once it has started and up to its conclusion (e.g., who will score next in a soccer match). It is also known as ‘live action’ betting and ‘in-running’ betting. Although it varies from sport to sport, live betting odds are essentially extracted from pre-match odds with (in the case of soccer) the current score, time remaining, and other elements all combined (e.g., the awarding of red and yellow cards, predicting next team or person to score, correct score, the total number of goals, etc.). Over one-quarter of all online gamblers in the UK have placed a bet in-play, with the largest proportion of those by those aged 25–34 years (Gambling Commission, 2018a, b, c). In-play betting is largely an online activity. *Bet365* (the most profitable online British bookmaker) reported that over three-quarters of their sports betting revenue is derived from in-play betting (Barber, 2018) and that the most popular sport to bet on is football (soccer).

As well as increased use of mobile device technology, there has been an escalation in the coverage of live soccer matches and other sporting fixtures from around the world. This has resulted in an expansion in the online betting market and an increase in the opportunities to bet on these in-play markets. This is set to continue to rise. The English soccer Premier League showed 200 out of 380 of its matches during 2018–2019, 42 more than 2017–2018 as a result of a new UK broadcast deal. These recent deals were the first time a full round of soccer matches were streamed live in the UK (BBC Sport, 2018). Subsequently, there will be an increase in matches for betting consumers to engage with.

Also of note is the ‘cash out’ facility that has been introduced alongside the ability to bet in-play. The ‘cash out’ feature is now offered to sports bettors by many online gambling operators. It enables sports bettors to settle an open bet for a value offered at the time of ‘cashing out’ (Lopez-Gonzalez & Griffiths, 2017). This figure is based on the current status of the bet and the statistical likelihood of the bet winning. This figure can also be higher or lower than the initial stake amount.

In recent years, increased attention has been given to researching in-play sports betting. A Gambling Commission (2016) prevalence survey reported that individuals who bet in-play were more likely to be categorized as problem gamblers. A recent scoping study identified 16 academic papers that had referenced in-play sports betting (Killick & Griffiths, 2019) and concluded that in-play sports betting has the potential to be more harmful than more traditional ways of gambling. The review also noted that different research methods had been used to explore this area. One method is the use of behavioral tracking data provided to researchers by online gambling operators. Such research has found that heavily involved gamblers are more likely to bet on in-play events (LaBrie, Laplante, Nelson, Schumann, & Shaffer, 2007) and that they increased the frequency of the number of in-play bets being placed after a three-month period (LaPlante, Schumann, LaBrie, & Shaffer, 2008). Secondly, some researchers have used self-report methodologies and reported an association between in-play sports betting and risk of problem gambling (Hing, Russell, Vitartas, & Lamont, 2015; Lopez-Gonzalez, Estévez, & Griffiths, 2019). The review also identified theoretical papers which had discussed the role of the structural characteristics of in-play sports betting. These papers argued that in-play betting had changed traditional sports betting from a discontinuous form of gambling into a more continuous one, and that the increased event frequency of in-play betting would be more likely to have an association with problem gambling than discontinuous (i.e., low event frequency) forms of gambling (Griffiths & Auer, 2013; Lopez-Gonzalez & Griffiths, 2017).

Two structural characteristics relevant to in-play sports betting and potential problem gambling are bet frequency (the number of bets placed in a particular time frame) and event frequency (how many games/matches are available to bet on in a certain period of time; Griffiths, 2012). It has also been argued that problem gambling is related to the structural characteristics that reinforce and facilitate gambling behaviour once it has started (e.g., bet frequency, event frequency, event duration, and pay-out interval; Griffiths & Auer, 2013). Lopez-Gonzalez and Griffiths (2017) suggested that the ‘cash out’ feature might be utilized during a time where emotions run high and the structural characteristics of this feature might facilitate sports bettors to lose control when they are placing their bets.

Lopez-Gonzalez et al. (2019) carried out a study of 659 Spanish sports bettors and examined the association between structural characteristics of online sports betting and gambling severity. The results demonstrated that sports bettors with high problem gambling scores were more likely to use in-play betting and the ‘cash out’ feature. More recently, Parke and Parke (2019) carried out in-depth interviews with 19 online problem gamblers. The core theme to emerge was labelled the ‘online sports betting loop’, which comprised the new structural features of the online sports betting market, and included in-play sports betting, cash out, and instant depositing. They noted that online sports betting offered features that allow gamblers to almost immediately re-engage with the sports betting activity. Some of their participants found it a challenge to maintain their self-control and others admitted chasing their losses. The authors suggested that attention should be directed towards increasing enforced breaks in this type of gambling.

Other studies on in-play sports betting have supported the idea that in-play sports betting may possess a number of features that encourage individuals to bet more, and there could be an association between in-play sports betting and a risk of harm from gambling (Lopez-Gonzalez et al., 2019; Lopez-Gonzalez, Griffiths, & Estévez, 2020; Parke & Parke, 2019). Lopez-Gonzalez et al. (2020) reported that within a sample of 659 Spanish sports bettors, those who engaged in-play sports betting (compared to those who did not) reported significantly greater (1) problem gambling severity, (2) sport watching consumption, (3) consumption of junk food, (4) alcohol consumption when watching sport, and (5) watching sport to escape from everyday preoccupations. They concluded that in-play betting was associated with impulsivity which occurred under circumstances where there was a high level of emotional involvement (i.e., watching live sport and betting on it).

A few studies have attempted to delineate the relationship between in-play sports betting and increased harm amongst problem gamblers. Previous research has found that impulse sports bettors prefer to bet in-play rather than on overall match outcomes (Hing, Russell, Li, & Vitartas, 2018). However, it is believed that trait impulsivity is not a unitary construct, but encompasses four individual traits: sensation seeking, lack of planning, lack of perseverance, and negative urgency (acting impulsively in the context of strong emotions; Sharma, Markon & Clake, 2014). Hing et al. (2018) suggested that research into contextual factors that contribute to urges to bet impulsively would help the field gain a better understanding of problematic gambling behaviour.

Another explanation that has been provided as to why sports betting may be associated with problem gambling is that betting features within live sporting events such as in-play betting and ‘cash out’ might make sports bettors more susceptible to experiencing cognitive biases (Lopez-Gonzalez, Estévez, & Griffiths, 2017; Lopez-Gonzalez & Griffiths 2017). Furthermore, technological advancements along with narratives found within sports betting adverts that enhance control could lead to an increase in perceived skill causing bettors to place their wagers more uncontrollably (Lopez-Gonzalez et al., 2017).

One cognitive heuristic related to gambling behavior is the illusion of control (Langer, 1975). The illusion of control is the inclination for individuals to overestimate the control they have over the outcome of events. It has been suggested that the illusion of control may be heightened because sports bettors can choose the amount to stake, the number of bets, and the speed in which they place them, which may result in sports bettors overestimating their control over uncountable events (Lopez-Gonzalez et al., 2017). The availability heuristic refers to the placing of more weight on information that is easier to recall. Information that is easier to recall is judged to be more common (Tversky & Kahneman, 1973) which leads to an overestimation of the probability of similar things happening in the future. Gamblers often utilize heuristics to process information more quickly such as representativeness heuristics. The use of these mental shortcuts could lead to biased decisions and/or distorted perceptions (Griffiths, 1994).

D’Astous and Gaspero (2015) reported that when there is a limited timeframe for bet placement, sports bettors (n = 161) used heuristic processing. Sports bettors were more likely to use heuristic (intuitive and fast) processing, rather than analytic processing (slow and deliberate). This form of processing was found to result in a lower gambling return on investment. Furthermore, this study reported more experienced gamblers were more likely to use analytic processing and their bets were more favorable (D’Astous & Gaspero, 2015). The authors suggested that these heuristic and analytic processes act as mediators in the relationship between previous experience and betting performance. It has also been argued that features such as in-play betting and “cash-out” betting may result in sports bettors having a higher likelihood of experiencing cognitive biases (Lopez-Gonzalez et al., 2017; Lopez-Gonzalez & Griffiths, 2017) and as a result place less planned bets.

Although the potential impact of emerging online sport betting features has been raised as a possible concern in relation to risk of problem gambling, to date, there has been minimal research carried out on the underlying mechanisms and attitudes towards specific online sports betting features including in-play sports betting and the use of the “cash out” feature. Consequently, the present study explored the opinions towards in-play sports betting behaviours. More specifically, it explored sports bettors’ perceived motivation and opinions towards online sports betting features. The specific objectives were to explore participants’ opinions and attitudes to: (1) in-play sports betting, and (2) towards the ‘cash out’ feature use within online sports betting.

## Methods

### Participants

The participants in the present study (n = 19) were recruited from across the UK including Nottingham, London, Bristol, Birmingham, Derby, York, Leeds, Sheffield, Oxford and Dundee (see Table 1). Participants comprised no-risk gamblers (n = 4), low risk gamblers (n = 7), moderate risk gamblers (n = 7), and one problem gambler (n = 1). Their ages ranged from 21 to 32 years (mean = 25.5 years; SD = 3.25). Of these gamblers, most were male (n = 17), and white ethnicity (n = 16). Two participants identified as mixed race and one was of non-white ethnicity. The education level ranged from general certificate of secondary education (GCSE) to bachelor degree level (12 participants had a degree). Over two-thirds of participants did not identify with a religion (n = 13), 13 participants worked full-time (68.4%), four were university students (21%), one worked part-time, and one was in the military. Seven participants were married or lived with their partners (36.8%) and one participant had children (see Table 1 for an overview of demographic characteristics).

**Table 1:** Basic demographic information of participants (n = 19)

Participant	Gender	Age	PGSI score
1	Male	30	7 (moderate)
2	Male	30	2 (low)
3	Male	26	5 (moderate)
4	Female	24	0 (no-risk)
5	Male	25	0 (no-risk)
6	Male	26	5 (moderate)
7	Male	29	2 (low)
8	Male	30	2 (low)
9	Male	24	4 (moderate)
10	Male	25	1 (no risk)
11	Male	30	0 (no-risk)
12	Male	30	1 (low)
13	Male	21	3 (moderate)
14	Male	31	13 (problem gambler)
15	Female	21	4 (moderate)
16	Male	24	2 (low)
17	Male	32	1 (low)
18	Male	26	2 (low)
19	Male	26	5 (moderate)

### Procedure

Qualitative interviews were conducted among a convenience sample of adults (n = 19) who had placed an in-play sports bet online within 6 months prior to the interview taking place. This inclusion criterion was selected to ensure that participants were able to freely discuss their experiences with in-play sports betting and it aimed to provide rich data. In order to recruit participants, members of the general public were approached outside of bookmakers in the research team’s home town, posters were put up around research team’s university premises, and via adverts on social media.

A semi-structured interview schedule was created using a number of open-ended questions which encompassed a set of key areas, previously identified within the literature. The interviews schedules included the following topic areas: (1) initial experience with sports betting, (2) experience of other gambling activities, (3) current sports betting behaviour (including in-play betting and use of the ‘cash out’ feature), (4) sports betting advertising, and (5) responsible gambling (full interview schedule available on request to the first author). Data were collected between August 2019 and December 2019. Participants were interviewed face-to-face at various locations including the research team’s university and the participant’s homes, apart from four interviews that were conducted via telephone. All interviews were recorded using a digital voice recorder, with consent from participants. Each participant was interviewed once (all of which lasted 25 min to 1 h). Demographic information in the form of a questionnaire was collected, including age, sex, occupation, marital status, highest level of education, marital status and city of residence (see Table 1).

### Data Analyses and Theoretical Approach

Interviews were recorded and analysed using NVivo, a qualitative data analysis computer software package. Thematic analysis was used in the present study because it is a flexible approach that delivers a rich and detailed, yet complex account of data (Braun & Clarke, 2006; King 2004). Six stages of thematic analyses were implemented, as outlined by Braun and Clarke (2006). These were: (1) familiarization of the data, (2) generating initial codes, (3) searching for themes, (4) reviewing themes, (5) defining and naming themes, and (6) producing the report. The themes emerged through careful reading and re-reading of the data (Rice & Ezzy, 1999). A general inductive approach to thematic analysis was adopted, whereby transcripts were read, re-read and coded line-by-line in order to identify key themes relating to the research aims. The researchers then met regularly to discuss the emergence of major themes. New prompts and areas for investigation were added to the interview schedule as they emerged. Themes were refined and any differences in interpretation were discussed until agreement was reached by the authors. These categories were organized into themes and sub-themes and findings are supported by direct quotations from the interviews in the “Results” section. Expressions are used to indicate approximate endorsement: ‘most’ (16 or more participants), ‘many’ (10–15 participants), ‘some’ (4–9 participants), and a ‘few’ (three or fewer participants).

The Problem Gambling Severity Index (PGSI) was used to assess problem gambling behaviour. Participants were asked to self-assess their gambling behavior and gambling consequences during the past 12 months across nine items. The results categorize the participant into one of the following groups: non-problem gambler, low-risk gambler, moderate-risk gambler, or problem gambler depending upon the score. The PGSI was used because it was specifically designed for the general population and has been found to be valid in calculating the degrees of problem gambling severity in a non-clinical context (Holtgraves, 2009). However, the PGSI groupings must be treated with some caution as they cannot be seen to sufficiently explain broader gambling behaviors for the participants. In the present study, two participants scored in the ‘moderate risk’ PGSI group, but also described patterns of excessive sports betting that were not picked up by the PGSI. Additionally, two participants said they had had gambled infrequently in the previous 12 months and scored in the ‘moderate risk’ PGSI group, but also described themselves as problem gamblers because of a previous ‘addiction’ to sports betting.

### Ethics

The research team obtained ethical approval from their university research committee. The participants signed a consent form, in which they were reassured that all their responses were confidential and anonymous and they had the right to withdraw from the study at any time. Additionally, participants gave their consent to be audio recorded.

## Results

Based on the analysis, the themes that emerged were categorized under the broad concepts of (1) accessibility of betting via a smartphone, (2) in-play betting motivating factors to participate, (3) in-play vs. pre-match betting engagement, and (4) beliefs and attitudes towards the ‘cash out’ feature.

### Accessibility of Betting via A Smartphone

The three sub-themes for betting via a smartphone were the: (1) transition from betting at a bookmaker’s shop, (2) ease in placing a bet, and (3) ability to place a bet anywhere.

#### Transition from Betting at A Bookmaker’s Shop

Many participants described how they initially began betting at a high street bookmakers’ shop, then transitioned to online gambling once it had become more popular. The factors that influenced sports bettors to gamble online included an increase in the number of online bookmakers, ‘welcome offers’ and other inducements offered by online operators, and the convenience of accessing online betting websites. Using a smartphone to place sports bets was the primary method of bet placement by everyone in the study sample. For example:

*“I guess I moved over from the bookies as soon as I had a [smart]phone for the first time”* (Participant 6).

*“We all went down on our lunch break to go and put football bets on. So I just started doing it then…in the shop on the coupon”* (Participant 17)

*“If I had a spare couple of quid I’d go and get bet slips down the bookies. I don’t go into bookies anymore, I do it all online; on apps and on the internet and stuff”* (Participant 12)

One participant commented that they preferred to place bets online because it offered a cash-out feature, whereas the high-street bookmaker did not:

*“Online betting gives you the option to cash out, and you don’t really get that ability in the betting shop”* (Participant 6)

#### Ease in Placing A Bet

Many participants commented on how easy it is to place a bet via a smartphone or tablet, compared to other methods (e.g., a laptop, high-street bookmakers). For example:

*“It’s obviously very convenient to do it on your [smart]phone or your tablet rather than getting a laptop out and logging in”* (Participant 5)

*“It’s just convenience, isn’t it? It’s in your pocket, turn it on [smartphone]. The apps are really easy to use”* (Participant 1)

Other participants commented that they bet on a mobile device due because it has the advantage of saving time. For example:

*“It’s easy to do – so it’s in front of you and it’s on your [smart]phone. There’s no going down to the [bookmaker] shops”* (Participant 12).

*“You could be out and about and think ‘there’s a couple of games later, I’ll just have a quick bet on it’ and then you’re away. You don’t have to scout around for a bookies or anything like that”* (Participant 18)

*“On an app, it literally is just the case of pressing buttons and pressing place bet…so it’s as quick as your thumb could move”* (Participant 17).

As well as gambling apps, there were other apps mentioned that were accessed on smartphones that assisted participants with bet placement. These were *Flash Scores* (a website that allows sports bettors to see live match updates) and *Odds Checker* (so sports bettors can compare odds against different online bookmakers). One participant discussed how using a smartphone allowed him to compare different inducements across gambling sites:

*“It’s normally on a mobile. It’s just easy isn’t it? You’ve got it to hand and you tend to get better offers online and you can see what offers they are straight away and compare them to other betting sites”* (Participant 2).

#### Ability to Place A Bet Anywhere

As previously mentioned, the most popular method for placing bets was on a smartphone. Some participants discussed how it was now possible for them to access the gambling apps in any location, at any time, and they did not have to rely on a laptop or computer in order to place a bet. Therefore, one of the main advantages of betting on a smartphone was the flexibility of location that it allowed. For example:

*“It’s really convenient being on a mobile [phone]. My phone’s in my hand the majority of the day anyway and the gambling apps are on my phone anyway”* (Participant 1).

Some participants discussed how they placed bets in multiple locations using their smartphones. Frequently mentioned betting locations included at the participant’s home, the pub, at friends’ houses, and at work.

*“It’s just easy to use. Use can use it when you’re at the pub, or in different environments. It’s handy for the in-play ones*” (Participant 19)

*“I liked having the convenience of being able to do it anywhere, anytime, not having to be at home sat in a specific place to do it. Which is probably part of the problem as well because I could literally do it anywhere. You know, I could do it in the car, out shopping, at work, anywhere like that and no-one would know what I was doing”* (Participant 5)

One participant commented on how he used his smartphone to check the status of his bet:

*“On a Saturday I would watch the scores live but if not I’m always checking my [smart]phone constantly. Last time I went out for a meal I had a bet running and everyone was like ‘why are you on your phone and I was like ‘the football’s on’”* (Participant 6).

### In-Play Betting Motivating Factors to Participate

The three main sub-themes as to reasons why participants engaged in live in-play betting were that it: (1) increases excitement, (2) makes the game more intense, and (3) allows gamblers to use their betting skill and knowledge.

#### Increases Excitement

Betting on a sporting event provided increased interest and excitement while watching it. Many participants commented that they took part in in-play sports betting because it increased their engagement with the game. This is because it made the game more exciting to watch because there was an opportunity for monetary gain. For example:

*“It increases excitement of that game and your attention and enjoyment”* (Participant 2).

*“It makes the game more interesting…and more exciting”* (Participant 11).

*“It’s quite fun trying to predict what’s going to happen”* (Participant 19).

One participant described online sports betting as a “buzz”. A feeling of excitement has come from placing a bet, and this is amplified if the bet is a winning bet, particularly if he is with friends and they are sharing the experience.

“*Well it’s just a bit of a buzz really. Like…if you’ve got a bet on something…the bets I tend to place are bets that like carry on going on for most of the game. Like…if I’m betting on an individual match, let’s say I’m betting on somebody to score at a particular time, you’ve got the whole game that could actually come through. So it’s the whole buzz and expectation thing. When you win it is actually a buzz, especially if you’re with your mates and they’ve got it on as well”* (Participant 8)

#### Makes The Game More Intense

Some participants discussed how in-play betting increased feeling of intensity when watching and betting on a match simultaneously. Sports betting on a match whilst watching it allowed for the game to be more psychologically interesting. For example:

*“It makes the game a more enjoyable and adds a bit of tension”* (Participant 19).

*“With in-play, you’re more invested in it. You can place sports bets that are in the future and if you do that, I don’t know about other people, but I can place a bet over a span of a few days and then forget about it and come back to it and think ‘oh, it lost’. Whereas in-play you’re more invested in what you’re watching anyway. So it’s not very often you would place an in-play sports bet and not be watching the play happen. There’s a bit more of a thrill to it I guess”* (Participant 3).

#### Allows Gamblers to Use Their Betting Skill and Knowledge

Some individuals engaged in in-play sports betting because they believed that they possessed skills which would influence the outcome of their bets, and thus providing them with a level of control. Many participants commented that they watched the game whilst betting on it, because this allowed them to assess the status of their match before placing a bet. For example:

*“If a team scores then they get momentum and they turn it over, especially if you’re expecting them to win anyway. So I suppose watching the game and thinking ‘yeah I know what’s going to happen’”* (Participant 6).

*“I guess in-play betting you feel more confident that you know. Everyone thinks they’re an expert. You’re watching it and you think ‘well there you go, this is actually quite accurate’…You’ve been watching the game and that would inform your decision”* (Participant 15).

*“If I’m not watching it because I can’t see what’s going on, I won’t [bet in-play]”* (Participant 7).

A few participants then discussed how a game developed and what they had observed during the match influenced them to place a bet. For example:

*“It might have been Newcastle [United] at Stoke [City] and I was watching it and I got the impression that Stoke were going to score before half time and they got a penalty in injury time in the first half and they scored and it felt great”* (Participant 7).

*“The ultimate goal is to beat the bookies, isn’t it? So I guess when you play in-play you think ‘I’ve analysed this and there’s loads of corners coming’, or God knows what. And you can make more of an educated guess. It makes you feel better about the gambling”* (Participant 11).

## In-Play Betting Vs. Pre-Match Engagement

Participants also made comparisons between fixed odds sports betting and in-play sports betting. Some of the participants commented on the dynamic odds being offered by online bookmakers during a game as a motivation for engaging in in-play sports betting. One of the benefits of this was possible monetary gains which were seen as an advantage. For example:

*“You can easily make more money in-play betting rather than pre-match betting because you know, with pre-match the odds are set at a certain price and that’s what the bookmakers offer. But in-play the price changes and that’s what a lot of people will look for. They’ll see whether they can get value. They'll see where they can maybe make as much money as they can”* (Participant 14).

*“Obviously the odds change as the match is going, so you can get quite lucrative winnings back depending on how much you put down”* (Participant 18).

*“I’ll only in-play bet if I’m actually watching the football because the idea, or the one good thing about in-play betting is that you can put a bet on before the match but you realize that ten minutes in that it’s not going the way that I thought it would and the team that I expected to win are actually not playing particularly well at all and it looks like the other teams are going to win and put on another bet”* (Participant 6).

Most participants discussed this in relation to football (soccer) because that was the event that they were betting on. However, one participant discussed this idea in the context of betting on tennis and darts:

*“You look at something that’s going against what should be happening and you try and hit it at the point when it’s furthest away from where it should be. I suppose tennis is a good example of that. The odds change so dramatically. [In] darts, the odds change so dramatically, that when you do it prior to the game you’ll never get odds on the favourite. But during the game, you can get great odds on someone like [tennis player Novak] Djokovic to beat someone outside of the top ten [tennis players] providing that Djokovic is already a set and a break down in the second [set] or something”* (Participant 12).

A few participants did view the odds changing as beneficial to their sports betting outcomes and preferred to place bets before the event started. This was because they had more time to think about the bet before they placed it. This was most popular for football (soccer) accumulators placed on Saturday fixtures. The following example is of a participant who wanted to take their time before placing the bet:

*“You have little time really, so you’ve got to rush yourself a little bit. I don’t like to rush myself, I like to think about it a little bit”* (Participant 10).

The timing of when the match was on was also discussed as a reason for placing a pre-match bet as opposed to an in-play sports bet. For example:

*“If it’s a Saturday, I’m more likely to do a pre-match bet because there is a full set of fixtures. If it’s on a weeknight for [the European soccer] Champions League or something like that, it’s more likely to be on TV, and I would be more likely to do an in-play bet when something’s happened in the game which triggers me thinking about betting”* (Participant seventeen).

*“But if I had 10 minutes, rather than….because bets are a bit more long term…like you can do kind of action…kinda sort of stuff…like with the in-play bets, you are like involved with it more often than not because you’re more invested in the game and whatever it is you’re sort of in-play betting with but if you start placing a bet on something like an accumulator and it’s got like a bet that’s on right now or something or starts like five hours later, you don’t really always keep track of it as much. But you can kind of place it and leave it*” (Participant 3)

The following participant discussed how the odds were something that changed rapidly:

*“The odds can change pretty fast. Obviously it just depends on what’s happening. So, if you’re betting on a football games that’s in-play and one team is obviously doing a lot better than the other, you can just start to see the odds getting shorter and shorter and shorter. If you place a bet sort of, fast, if you hesitate a little bit maybe, you could end up, if it wins, obviously if you place money on that bet you’d get less money than you thought you would with the bet had you been a little bit quicker placing it. That’s just in-play. It’s just the environment with it I guess”* (Participant 3).

A few participants reported that in-play sports betting allowed them to continue their betting and allowed them an attempt to recoup their losses or place multiple bets in a game. For example:

*“It’s almost like an instant win depending on what you’re in-play betting on. You don’t have to wait until the end of the game to win so you could potentially have a few in-play bets on the same game”* (Participant 7).

*“It does make it very easy or very quick to put bets on in a short space of time, and I always think that that’s kind of dangerous if you do get in that mindset”* (Participant 9).

### Beliefs and Attitudes Towards The ‘Cash Out’ Feature

There were three sub-themes related to the ‘cash out’ feature: (1) recouping a losing bet, (2) the ‘cash out’ monetary value being high, and (3) regret after cashing out.

#### Recouping A Losing Bet

The analysis showed that participants had different motivations for cashing out, including minimizing losses when the bets were losing, and acquiring more funds to allow the placement of additional bets. Bets can be withdrawn whilst a sporting event is still in play, to guarantee at least some profit and/or to minimize losses. This was dependent upon the cash out value being of what the participants perceived to be an acceptable amount. Some participants chose not to cash out their bets at all. All participants had cashed out a bet at some point in their life. The most popular sport where the cashing out of bets was during football (soccer) matches. Reasons for this included the length of the sport (i.e., being a 90-min game), and there were more likely to be surprises or changes within the game which resulted in the participant potentially cashing out their bet. One participant talked about how once the game started and they cashed out, the newly acquired funds allowed them to gain momentum and continue betting to reach an expected target that they originally had in mind. For example:

*“I think it encourages quite a bit of repetition betting in a sense that you might cash out and use that money straight away [to re-bet]. So there’s a bit more of a momentum type thing. If you cash out, and say you’ve got a bet with returns of £420 and you cash out at £70 and you’ve only placed £10 down to get that, you’re still £60 up. But you want that, or you have an idea of £420 in your head at some point. So you think ‘I’ve got a bit of a bigger sum to reach here-so you’ll probably just invest your money back into the site’. I think at that point, once you’ve got something, I’m not playing with the original money that I invested with anymore”* (Participant 3).

Another participant discussed the emotions that came into play when deciding whether or not to cash out their bet. They described different emotions they have experienced, with one way resulting in cashing out the bet due to “nerves” and the other letting the bet ride because they felt more confident. For example:

*“I would cash out because of the nerves. I’d be sweating it, thinking ‘you know I’ve made the money, let’s not be greedy, have it over and done with’. But on the other hand I haven’t cashed out because I’m confident, I’m risking it a little bit”* (Participant 16).

*“I’ve put on an unlikely bet and my team has scored but they’re still losing so they’ve offered me a cash out which was more than my stake and I’ve changed my mind and thought the team I bet on aren’t going to win, so it’s worth taking the extra or doubling my stake instead of ten times my stake. Or just cutting my losses essentially”* (Participant 17).

#### The ‘Cash Out’ Monetary Value Being High

The cash out value has to be enough to be deemed worth cashing out by the sports bettor. For example, in the instance that the bet has made a profit on the initial stake. The performance of the team may influence whether individuals cash out their bet because they feel the team are not performing well and the bet may lose. For example:

*“If I thought that the bet was going to lose then you want to try and recover as much of the original stake as possible”* (Participant 19).

*“If the accumulator has got to a good amount where I’m making at least more money than I expected or if they look like…I don’t think the team is going to win or the bet isn’t going to come in then I’ll try and cash out early but usually I’ll end up just leaving [the bet] on”* (Participant 11).

Other participants would only cash out if they were betting for a profit, or alternatively if they thought that the cash out value was at an amount that was worth taking. For example:

*“It will always be for a profit. If I cashed out and lost money, I’d think well I might as well have let it run its course”* (Participant 7).

*“I probably would cash out if my winnings were say £250 to £300, because that’s quite a lot of money. Say I’m on for £15/£20 I’d probably just see [the bet] through until the end”* (Participant 18).

#### Regret After Cashing Out

A few participants expressed that they had not always made the correct cash out decision and then came to regret it after. This went both ways with participants either affecting their profits by taking a risk that turned out to be cashing out too early, rather than letting the bet ride or have the bet lose and not cash out. For example:

*“I’ve won a few bets but cashed out too early so I just don’t bother anymore”* (Participant 19)

*“There’s been a lot of times that I’ve waited on it and thought ‘no, I’m going to ride it out’ and it’s lost and I should have taken the cash out”* (Participant 6).

*“There’s been a few times where I haven’t cashed out and I’ve regretted it because I’ve been close to winning money and I’ve been offered quite a good amount of money to cash out and I’ve not taken that option”* (Participant 10).

*“A couple of weeks ago I placed an in-play accumulative. It was like five teams and within about 20 minutes in the second half they were all winning…I was getting offered £90 but had I let it run. I was being offered around £400 but I took it anyway. I took the £90 quid and of course 90 minutes came and if I'd let it play, it would have won, but I still see it as making money anyway. I still made £70 but I was a bit hacked off. Had I just let it run…I could have had a bit more…I'm still not been able to decide whether cash out is a good thing or not because it can be beneficial at some point and I guess that's the risk you take”* (Participant 14).

## Problem Gambling Behavior and In-Play Betting Features

Whilst most participants discussed the advantages of in-play betting on a mobile app, there were some aspects of mobile betting that appeared to encourage problematic gambling behavior. Online sports betting removes the social context where people who have problems with their gambling behavior might experience guilt, self-consciousness, fear of stigma, and friend or family intervention due to repeated losses and high expenditure. For example, the following participants who had experienced gambling problems discussed how they were more likely to remain in control of their in-play betting expenditure when they were in a social environment:

*“There’s been times when I’m with friends, I’ll make bets that are always a bit lower but when I’m on my own I have a moment of ‘you know what, I’m going to put a big bet on this’ just because, like, no-one’s around to be like ‘don’t do that’”* (Participant 1)

One participant described how sports betting was initially a social activity that then developed into a more compulsive behavior, associated with secrecy. Betting on a mobile allowed this individual to hide their gambling whilst in the same room as their partner:

*“It kind of got more of a problem when I started sports betting and I would do it on my own. Or we might be in the same room on the sofa but I’d be on my phone and [my partner] wouldn’t know what I was doing. I was very secretive about it once it got past that initial gambling for fun stage”* (Participant 5)

Mobile betting provides a solitary environment and appears to facilitate riskier gambling in these cases compared to in-person betting at a high street bookmaker. This was reflected in the some of the quotes:

*“I liked having the convenience of being able to do it anywhere, anytime, not having to be at home sat in a specific place to do it. Which is probably part of the problem as well because I could literally do it anywhere. You know, I could do it in the car, out shopping, at work, anywhere like that and no-one would know what I was doing. It helped me to keep it secret…even if I was at the gym I’d have my phone with me and I’d be able to place bets at the gym and follow them whilst I was there. Anywhere really, anywhere that I could get a bit of privacy so no one could see what I was doing”* (Participant 5)

*“I've been gambling for so long and you know, addiction has cropped up quite often and I kind of keep it private now. Well, as much as I can. So, like no-one really knows that I do it anymore. Well, they do and they don’t. Sometimes I just can’t hide it, especially if I had like a big loss, people know. I do it on my tablet and I do it in private because, one, I don’t want anyone complaining at me, and two, I don't want anyone getting worried. Um, three, it's a personal thing. I want to enjoy it myself. It sounds a bit morbid actually, you know, now that I'm talking about it, but yeah, I do it on my tablet and I do it alone. I never gamble with friends”* (Participant 14)

In most cases, participants described online sports betting as a gambling activity involving skill, analysis, and engagement with the sporting event. However, one participant described how as their gambling behavior became increasingly problematic, they transitioned into placing bets without much thought of the outcome and without prior analysis, but instead on the type of bet that would get them the highest monetary return:

*“On a roulette table or blackjack there is a house edge and will lose eventually no matter how good of a run you go on because they’re designed that way. But with sports betting, I felt like I could analyze the form and look at the game and get a feel for it and bet according to that, which is what I first started betting on. But as it got later on, I wasn’t betting based on any data, or form, or feeling. I was literally just looking at the odds and placing a bet on it. I was betting on anything…that I maybe had no idea about”* (Participant 5)

There is a constant stream of sports betting opportunities available for in-play betting. One participant described how they temporarily banned themselves from gambling online after they began in-play betting on sporting events that they would not ordinarily be interested in:

*“There was probably a little bit of an addictive sort of temptation, like, looking at your phone and placing bets on matches that you didn’t really care about”* (Participant 19)

## Discussion

The purpose of the present study was to contribute to knowledge concerning online sports betting features, specifically in-play sports betting and the ‘cash out’ feature. Based on the analysis, four broad themes emerged (accessibility of betting via a smartphone, in-play betting motivating factors to participate, in-play vs. pre-match betting engagement, and beliefs and attitudes towards the ‘cash out feature) comprising nine subthemes (see Table 2).

**Table 2:** Table of themes and sub-themes from interviews with online sports bettors (n = 19)

Theme	Sub-theme
Accessibility of betting via a smartphone	Transition from betting at a bookmaker’s shop
	Ease in placing a bet
	Ability to place a bet anywhere
In-play betting motivating factors to participate	Increases excitement
	Makes games more intense
	Allows gamblers to use their betting skill and knowledge
In-play vs. pre-match betting engagement	
Beliefs and attitudes towards the ‘cash out’ feature	Recouping a losing bet The ‘cash out’ monetary value being high Regret after cashing out

To date, there has been a small amount of research carried out on in-play sports betting and the findings from the present study will be discussed in relation to these. One theme was ease of access to in-play sports betting using smartphones. Sports bettors now have immediate access to sports betting websites in most locations and situations. The findings here suggest that smartphone betting allows immediate access to gambling, supporting previous research that online gambling is easy to access via mobile devices (Deans, Thomas, Daube, & Derevensky, 2016). It has been previously suggested that this increased accessibility to online gambling websites and the ease of being able to use online platforms, may speed up maladaptive learned behaviours, including problem gambling (James, O'Malley, & Tunney, 2016; McCormack & Griffiths, 2013). The sports bettors within the present sample had a preference for placing bets on mobile devices, which supports previous research that sports bettors (83.4%) prefer to use a remote device to place a bet rather than going to a betting shop (Lopez-Gonzalez et al., 2019). Additionally, the same study found that problem gamblers were more likely to prefer to use a mobile device.

Sports bettors in the present study would often bet on the match in order to make the game appear more exciting and intense. Previous research has suggested that one way in which the structural characteristics of in-play sports betting that may contribute to problem gambling is that they make the event more interesting and/or exciting (Parke & Griffiths, 2007). Gambling games that involve speed and excitement have been previously associated with problem gambling (Parke & Griffiths, 2007). Whilst the findings here concur with previous research, it may be of value to investigate to clarify which features of in-play sports betting add to the excitement and if these are more specifically related to problem gambling.

One area that was prominent within the interviews was participants’ awareness of the odds that were being offered during in-play betting by the bookmakers. Lamont, Hing and Vitartas (2016) reported that live odds updates during sports events may prompt bettors into placing impulse bets. These impulse bets were more likely to be placed if the odds were perceived as good and related to their favourite team. Some participants believed that it was easier to make money in in-play sports betting as opposed to betting before a match. Some gamblers perceive sports betting as a skill-based form of gambling (Cantinotti, Ladouceur & Jacques, 2004). Previous research on motivations to engage in sports betting in Tasmania (Australia) was related to the sports bettor’s perceived amount of knowledge or experience with the sports that they were betting on (Palmer, 2014). In a study of 258 individuals, Khazaal et al. (2012) reported that experts (i.e., professional soccer players, coaches, or journalists) were no more successful at predicting soccer match outcomes than the non-professionals. In systematic review carried out on the role of chance and skill in sports bettors (and focusing on cognitions in the behaviour), Mercier et al. (2018) reported that sports bettors overestimated the importance of skill on the overall match outcome. Ladouceur, Giroux and Jacques (1998) found that gamblers on horse races who were classed as ‘experts’ picked more winning selections but did not have better monetary outcomes than random selection. It was concluded that the experts were more thoughtful, careful, and likely to place safer bets.

It is possible that some in-play sports gamblers may experience higher levels of perceived skill in the activity due to cognitive distortions. It is possible that cognitive distortions could lead to the development and maintenance of a gambling disorder. Cantinotti, Ladouceur and Jacques (2004) examined whether the idea of having betting skill was illusion or chance. Compared to bets made by expert sports bettors versus randomly selected wagers, they found that sports bettors demonstrated a higher accuracy for correctly predicting game outcomes compared to chance (i.e. randomly selected bets), although, the overall amount of money won was not higher than chance. The researchers concluded that the notion of skill when betting is the result of cognitive distortions.

Theoretical papers that have focused on the structural characteristics of in-play sports bettors have specifically noted that the nature of the gambling activities has changed from what was previously a discontinuous (low-risk) form of gambling to a continuous (high-risk) form of gambling (Griffiths & Auer, 2013; Lopez-Gonzalez & Griffiths, 2017). In the present study, participants noted that getting a bet credited in in-play sports betting felt like *“an instant win”* and that multiple bets could be placed within a small window. Therefore, the shortening of bets being paid out has reduced delays in receiving rewards from gambling, and allowing the gambler the potential of placing multiple bets per match.

The present study found that reasons for using the ‘cash out’ feature varied between individuals. Some individuals cashed out to cut their losses, whilst others cashed out when they were betting a profit, while other preferred not to cash out and let the game run to completion. Lopez-Gonzalez et al. (2019) found that problem gamblers were more likely to use the ‘cash out’ feature than non-problem gamblers. Further research should investigate what types of individuals (in terms of demographics and personality traits) use the ‘cash out’ feature and their motivations for doing so. Comparisons have previously been made between the ‘cash out’ feature and stock market trading (Lopez-Gonzalez & Griffiths, 2017). For example, cashing out is similar to a stop-loss order within financial trading, which is an order to sell an existing shareholding which is triggered if the bid price falls to, or below the stop price set by a trader. This might be used when somebody buys a share to give them some protection and help minimize loss should a share price fall. With the ‘cash out’ feature, individuals can decide whether they are going to cash out when it receives a specific level of profit or cash out when the bet is losing a specific amount (or alternatively let the bet run until the end). Lopez-Gonzalez and Griffiths (2017) claimed that in-play online sports betting may benefit from regulations that are currently applied within the stock market industry.

## Limitations

The present study has a number of limitations to take into account when interpreting the findings. Firstly, the sample mainly consisted of non-problem male sports bettors, despite efforts by the research team to recruit female gamblers and more problem gamblers. Future research should attempt to recruit greater numbers of females and problem gamblers in their samples. Secondly, the present study specifically targeted individuals who had placed at least one sports bet within the past six months. For this reason, participants may have had varied levels of engagement with sports betting and although they were assumed to qualify and meet the aims of the study, they were not representative of all online sports bettors or the wider betting population. Thirdly, the study relied on self-report data which can be affected by a number of well-known biases (such as social desirability and recall biases). Finally, it is important to highlight that the study was exploratory which allows for a preliminary understanding of in-play sports betting behavior, rather than allowing for definitive conclusions, especially because of the sample size.

## Conclusion

Overall the sports bettors in the present study viewed in-play sports betting favorably and readily accessible. However, the results demonstrated that this is a way of gambling that can be played without interruption and which may lead to repetitive (i.e., continuous) gambling and/or unwarranted feelings of control. Given that this was an exploratory study, further research is required in order to draw more definitive conclusions. Future research could focus on the following areas: (1) qualitative and quantitative studies examining the motivation and perceptions of in-play sports betting use with females and/or samples of vulnerable individuals; (2) empirical studies on how factors such as the marketing and advertising of sports betting products influence sports betting behavior; and (3) longitudinal studies to track the game-play of in-play sports bettors. Further research into this area is required in order to provide direction for policymakers to develop responsible gambling measures for this relatively new way of gambling.
